# Surgical Efficiencies and Quality in the Performance of Voluntary Medical Male Circumcision (VMMC) Procedures in Kenya, South Africa, Tanzania, and Zimbabwe

**DOI:** 10.1371/journal.pone.0084271

**Published:** 2014-05-06

**Authors:** Dino Rech, Jane T. Bertrand, Nicholas Thomas, Margaret Farrell, Jason Reed, Sasha Frade, Christopher Samkange, Walter Obiero, Kawango Agot, Hally Mahler, Delivette Castor, Emmanuel Njeuhmeli

**Affiliations:** 1 The Centre for HIV and AIDS Prevention Studies, Johannesburg, South Africa; 2 Tulane School of Public Health and Tropical Medicine, Department of Global Health Systems and Development, New Orleans, Louisiana, United States of America; 3 U.S. Department of State, President’s Emergency Plan for AIDS Relief, Washington, D.C, United States of America; 4 Institute of Continuing Health Education, University of Zimbabwe, College of Health Sciences, Harare, Zimbabwe; 5 National Reproductive Health Society, Kisumu, Kenya; 6 Impact Research and Development Organization, Kisumu, Kenya; 7 Jhpiego, Dar es Salaam, Tanzania; 8 United States Agency for International Development, Washington D.C., United States of America; World Health Organization, Switzerland

## Abstract

**Introduction:**

This analysis explores the association between elements of surgical efficiency in voluntary medical male circumcision (VMMC), quality of surgical technique, and the amount of time required to conduct VMMC procedures in actual field settings. Efficiency outcomes are defined in terms of the primary provider’s time with the client (PPTC) and total elapsed operating time (TEOT).

**Methods:**

Two serial cross-sectional surveys of VMMC sites were conducted in Kenya, Republic of South Africa, Tanzania and Zimbabwe in 2011 and 2012. Trained clinicians observed quality of surgical technique and timed 9 steps in the VMMC procedure. Four elements of efficiency (task-shifting, task-sharing [of suturing], rotation among multiple surgical beds, and use of electrocautery) and quality of surgical technique were assessed as explanatory variables. Mann Whitney and Kruskal Wallis tests were used in the bivariate analysis and linear regression models for the multivariate analyses to test the relationship between these five explanatory variables and two outcomes: PPTC and TEOT. The VMMC procedure TEOT and PPTC averaged 23–25 minutes and 6–15 minutes, respectively, across the four countries and two years. The data showed time savings from task-sharing in suturing and use of electrocautery in South Africa and Zimbabwe (where task-shifting is not authorized). After adjusting for confounders, results demonstrated that having a secondary provider complete suturing and use of electrocautery reduced PPTC. Factors related to TEOT varied by country and year, but task-sharing of suturing and/or electrocautery were significant in two countries. Quality of surgical technique was not significantly related to PPTC or TEOT, except for South Africa in 2012 where higher quality was associated with lower TEOT.

**Conclusions:**

SYMMACS data confirm the efficiency benefits of task-sharing of suturing and use of electrocautery for decreasing TEOT. Reduced TEOT and PPTC in high volume setting did not result in decreased quality of surgical care.

## Introduction

Evidence from three clinical trials has demonstrated that medical male circumcision reduces HIV transmission in heterosexual men by approximately 60% [1]–[3]. Data from mathematical modeling indicate that achieving 80% voluntary medical male circumcision (VMMC) coverage among men ages 15–49 years in 14 Eastern and Southern African countries could avert 3.4 million new HIV infections by 2015. However, to reach this goal, it would be necessary to perform 20.3 million circumcisions by 2015 [4]. Starting in 2008, governments, technical agencies, providers, and international donors began work on the scale-up [5]–[7]. However, programs are unlikely to meet this goal by 2015 [8].

Little precedent exists for delivering a surgical intervention for public health impact at this scale. Aravind Eye Clinic in Madurai, India, pioneered several efficiencies for cataract surgeries, transferable to VMMC: (1) task-shifting (using paramedical personnel to perform repetitive surgeries), (2) task-sharing (of time-consuming tasks such as patient preparation), (3) pre-bundling of instruments prior to surgery, and (4) use of multiple surgical bays. These practices increased output ten-fold, while maintaining quality of care [9]. In Mozambique task-shifting of obstetric care to specially trained assistant medical officers increased coverage of obstetrical services, with outcomes comparable to obstetricians, even among cases with complications [10], [11]. Curran et al. [12] identified task-shifting and task-sharing as critical to the successful VMMC scale-up in Kenya.

To assist countries seeking to scale-up VMMC, a World Health Organization (WHO) panel issued “Models for Optimizing the Volume and Efficiency for Male Circumcision Services [13], which outlined “considerations” for improving efficiency while ensuring safety, given local context and national policy. Practitioners working with the scale-up identified six elements to enhance surgical efficiency in high volume settings: use of task-shifting, task-sharing, pre-bundled kits with disposable supplies, rotation among surgical beds, electrocautery, and forceps-guided surgical method. (For extent of adoption of these elements, see [14]).

The objectives of this analysis were (1) to document the time required to complete each step in the VMMC procedure, (2) to test four elements of surgical efficiency in relation to two outcome measures: the time the primary provider spends with the client (PPTC) and the total elapsed operating time (TEOT), and (3) to test the association between surgical quality and these two outcome measures. Although efficiency is critical to other activities that form part of comprehensive VMMC services (e.g., STI diagnosis and treatment, HIV counseling), this research focused specifically on efficiency related to performing the surgical procedure.

## Methods

The methods for the Systematic Monitoring of the Voluntary Medical Male Circumcision Scale-up (SYMMACS) are described elsewhere [14]. Two serial cross-sectional surveys of VMMC sites were conducted in Kenya (KE), Republic of South Africa (RSA), Tanzania (TZ) and Zimbabwe (ZW) in 2011 and 2012. A clinician trained in VMMC observed up to 10 VMMC procedures per site to assess the quality of the surgical techniques used and timed nine steps in the procedure (listed in Table 1). The steps were (1) client’s entrance, (2) scrubbing skin, preparing foreskin, (3) administering local anesthesia, (4) removing foreskin, (5) performing haemostasis using electrocautery or ligating sutures, (6) inserting skin sutures (primary provider), (7) inserting skin sutures (secondary provider, if applicable), (8) cleaning client, applying dressing, and (9) client’s exit. In RSA and ZW, the primary provider was almost always a medical doctor who at a minimum completed the most clinically complex steps (#4–6): removing the foreskin, achieving haemostasis, and inserting the mattress sutures. In KE and TZ, other trained clinical providers (nurses, clinical officers, or assistant medical officers, depending on country) were authorized to perform all nine steps.

**Table 1:** Median time (in minutes: seconds) per step in the VMMC procedure, primary provider time with client (PPTC), and total elapsed operating time (TEOT) by country and by year.

	Kenya		South Africa			Tanzania				Zimbabwe	
	2011	2012	2011	2012		2011	2012		2011		2012
	n = 151	n = 218	n = 120	n = 361		n = 126	n = 251		n = 140		n = 204
**1.** Patient enters operating area	7∶02	6∶12	4∶03		3∶35	2∶24		2∶24		2∶00	2∶20
**2.** Provider scrubs & prepares patient skin	1∶14	1∶02	0∶51		0∶40	1∶32		1∶38		1∶10	1∶08
**3.** Provider administers local anesthesia	1∶07	1∶00	0∶49		0∶57	0∶57		0∶57		1∶26	1∶10
**4.** Provider removes foreskin	1∶06	1∶12	0∶04		0∶45	1∶30		1∶18		0∶49	0∶03
**5.** Provider performs hemostasis using:											
a. electrocautery	*–*	*–*	2∶07		2∶33	*–*		*–*		1∶44	2∶00
b. ligating sutures	4∶05	3∶56	–		3∶51	5∶18		5∶44		3∶08	4∶16
**6.** Primary provider inserts skin sutures	8∶15	7∶26	3∶23		2∶52	6∶55		7∶11		4∶40	3∶28
**7.** Secondary provider assists with insertion of skin sutures	–+	–	4∶59		4∶31	5∶51		2∶59		4∶24	4∶56
**8.** Provider applies dressing & cleans the client	1∶15	1∶16	1∶41		1∶44	2∶50		2∶31		2∶00	2∶11
**9.** Client dresses and exits operating theater	0∶22	0∶21	2∶25		2∶19	0∶46		0∶34		1∶05	0∶45
**Primary provider time with client (PPTC)**	13∶34	12∶53	6∶19		6∶45	14∶06		14∶47		7∶58	6∶48
**Total elapsed operating time (TEOT)**	22∶42	23∶10	23∶31		29∶41	24∶41		23∶12		27∶20	23∶20

+ Data omitted (one case only).

We tested four elements of surgical efficiency in relation to two outcome variables: PPTC and TEOT. The four elements were (1) task-shifting (measured by cadre serving as primary provider); (2) task-sharing (secondary provider assists primary provider in completing suturing), (3) rotation among multiple beds (measured by mean number of beds in use during observation); and (4) use of electrocautery to achieve haemostasis. Two additional efficiency elements were excluded from this analysis. Forceps guided surgical method had become nearly universal by 2012 in all countries; use of prepackaged kits was measured at the site level, not during observation of the VMMC procedure.

The association between quality of surgical technique and each outcome variable (PPTC and TEOT) was tested. Quality was assessed using a 13-item checklist, drawn from WHO guidelines for quality assessment [15]. The 13 items were to clean surgical area with a recommended scrub solution, correctly identify the skin to be excised, demonstrate the “safety first approach” to ensure no part of penis besides the foreskin is in danger of being injured, demonstrate safe administration of local anesthesia, demonstrate cautious & gentle approach to removing the foreskin, adequately controls bleeding with electrocautery and/or ligating sutures, use correct technique to tie surgical knots, correctly align the frenulum and places secure mattress suture, correctly align the other quadrant sutures, avoid placing deep sutures around the frenulum, place interrupted sutures evenly to avoid leaving gapping margins, ensure no significant bleeding present, and place a secure dressing that is not excessively tight. SYMMACS clinicians scored each item as 0 (unsatisfactory), 1 (partially satisfactory), and 2 (satisfactory), based on written criteria. Each score of 2 contributed one point toward a 13-item scale for a possible score of 0–13. Given that most providers scored 12–13, the data were collapsed as<12, 12 and 13 in the bivariate analysis.

PPTC was measured as the time required to complete steps #4, 5, and 6: remove foreskin, achieve haemostatis, and insert mattress sutures. Where task-shifting is not authorized, these steps must be completed by the doctor. TEOT was measured as steps 2–8, excluding entry and exit; it included “down-time” between steps (e.g., waiting for the primary provider to become available). TEOT directly influences the number of procedures that a team can complete on a given day.

The data were analyzed using SPSS version 19. Attempts to normalize the data via natural log and square root transformations were unsuccessful. The data on timing of each step, PPTC and TEOT were non-normally distributed across all countries and years, and positively skewed. For the bivariate analysis Mann Whitney tests were used for all dichotomous variables (task-shifting, task-sharing, and electrocautery). Kruskal Wallis tests were used for variables with more than two categories (number of beds and quality score). Mann Whitney was also used to test for differences between years within country; Kruskal Wallace to test for differences among countries for a given year. The relationship between quality score (treated as a continuous variable) and TEOT was tested using Spearman rank correlations. P-values less than 0.05 were termed significant (unless stated otherwise).

The original data analysis plan called for multivariate analysis combining data across the four countries. However, utilization of efficiency elements depends on national policy, with minimal in-country variation; the country effect (collinearity between country and efficiency element) precluded multivariate analysis of a combined data set. Instead, linear regression analysis was performed separately for each country. Task-shifting, task-sharing, and type of hemostasis were analyzed as dichotomous variables with a reference group in the regression; number of beds and quality score were treated as interval variables. No controls were included in the modeling. Variables with less than 5 cases per cell were excluded from this analysis; as a result, only number of beds and quality score appeared in the regression models for all countries and years.

Given the dearth of literature on operational aspects of VMMC, the researchers opted to focus narrowly on efficiencies in the surgical performance of VMMC rather than the wider range of activities that comprise comprehensive services or to include such factors as client waiting time.

All study participants provided written consent and the consent forms were all approved by various IRB. Human subject approval was obtained through the Tulane University Institutional Review Board (IRB) and the local IRBs in each country; the Kenya Medical Research Institute, University of the Witwatersrand’s Human Research Ethics Committee in South Africa, Tanzanian National Institute for Medical Research, and the Medical Research Council of Zimbabwe. All those above-mentioned IRB approved the full study.

## Results

Timing was estimated from VMMC procedures observed in 2011 (N = 537) and 2012 (N = 1,034) across the four countries. By country and by year, the number of VMMC procedures observed was: KE (N_2011_ = 151 and N_2012_ = 218), RSA (N_2011_ = 120 and N_2012_ = 361), TZ (N_2011_ = 126 and N_2012_ = 251) and ZW (N_2011_ = 140 and N_2012_ = 204). Table 1 shows the median time for each of nine steps in the procedure, by country and by year. Significant variations existed across countries on all steps (p<0.001) except#7. However, the most marked variation involved step#5: achieving haemostasis. The median time ranged from 1∶44 to 2∶33 minutes across countries for providers using electrocautery, compared to 3∶08 to 5∶18 minutes for providers using ligating sutures – a difference of 3∶49 minutes (based on averaging the difference in each country in each year). Significant differences also occurred for inserting skin sutures. In RSA and ZW the primary provider inserted the mattress sutures (step#6) but a secondary provider completed the suturing (step #7). The total suturing (both steps) was lower in RSA and ZW than in KE and TZ. Considering step#6 alone, in RSA and ZW, suturing time was minimized by 4∶47 minutes by having a secondary provider take over and complete suturing after insertion of mattress sutures. However, suturing time may also reflect the median number of sutures applied: 12 in KE and TZ, 10 in RSA and ZW.

Table 1 presents the two outcome measures of efficiency: PPTC and TEOT. The median time for PPTC was significantly higher in KE and TZ (between 12∶53 and 14∶47 minutes in the two countries over the two years of the survey) than in RSA and ZW (between 6∶19 and 7∶58 minutes) (p-value<0.001). Three factors explain this difference: (1) the average time-saving of over two minutes from using electrocautery, and (2) the use of a secondary provider to complete suturing after the doctor inserted the mattress sutures, and (3) fewer sutures inserted (10 versus 12). By contrast, in the two countries where other clinical providers (not doctors) performed the full operation (KE and TZ), the provider was more likely to complete all suturing and stay with a client throughout the whole operation.

The median TEOT ranged from 22∶42 to 29∶41 minutes in the four countries over the two years. Six of the eight data points (4 countries×2 years) shown in Table 1 fell between 22∶42 minutes and 24∶41 minutes, suggesting 23–25 minutes as the modal TEOT (steps #2–8) across the four countries. The median TEOT differed across countries in both 2011 and 2012 (p<0.001). KE had the shortest TEOT in both years; the longest TEOT corresponded to ZW (2011) and RSA (2012).

The shorter PPTC in RSA and ZW did not reduce the TEOT, as compared to KE and TZ, for two reasons. First, the time used by the secondary providers to complete the suturing was factored back into the TEOT. Second, use of multiple surgical bays may have increased wait time for clients in the surgical cubicles, as the primary provider moved between beds.

The second part of the analysis tested for an association between five explanatory variables – four elements of surgical efficiency and surgical quality– and two efficiency outcome variables: PPTC and TEOT. Table 2 shows the descriptive statistics for the five explanatory variables. These descriptive data reiterate the point made in the overview article [16], that utilization of the efficiency elements differed significantly by country, dictated largely by national policies; there was very little in-country variation. Specifically, task-shifting was authorized in KE and TZ, but not RSA and ZW. Table 2 also provides data on task-sharing (the division of labor regarding suturing). Where task-shifting was authorized, the primary provider (not a medical doctor) tended to complete all suturing: in KE, 99% of all procedures in 2011 and 100% in 2012; in TZ, 84% in 2011, 97% in 2012. By contrast, in RSA the primary provider performed all suturing in only 40% of procedures in 2011 and 0% in 2012. Zimbabwe was more mixed, with the primary provider completing the suturing alone in 57% of cases (2011) and 46% of cases (2012). Electrocautery was universal in RSA (at least 99% of the cases in both years) and was on the increase in Zimbabwe, but not used in KE and TZ. Regarding rotation among beds, in RSA and ZW over 70% of procedures conducted in 2011 occurred in sites with more than four beds. In TZ, over two-thirds of procedures were performed in sites with 2–3 beds. By contrast, use of multiple beds was least common in KE.

**Table 2:** Descriptive statistics on four surgical efficiency elements and the quality score for VMMC procedures, by year and by country**.**

	Kenya				South Africa				Tanzania				Zimbabwe			
	2011		2012		2011		2012		2011		2012		2011		2012	
	n	%	n	%	n	%	n	%	n	%	n	%	n	%	n	%
**Task-shifting: cadre of primary provider**																
Physician	0	0%	1	0%	119	99%	355	99%	12	9%	12	5%	139	100%	202	100%
Non-physician	151	100%	216	100%	1	1%	4	1%	116	91%	239	95%	0	0%	0	0%
**Task-sharing: who performed the suturing: primary provider (alone) or primary and secondary provider (both)**																
Alone	150	99%	218	100%	48	40%	0	0%	106	84%	243	97%	80	57%	110	46%
Both	1	1%	0	0%	72	60%	361	100%	23	16%	8	3%	60	43%	130	54%
**Mean number of beds**																
1	85	56%	158	73%	8	7%	35	10%	42	32%	54	23%	0	0%	0	0%
2–3	55	36%	60	27%	20	17%	115	32%	91	68%	164	69%	40	29%	142	70%
4+	11	7%	0	0%	92	77%	211	58%	0	0%	20	8%	100	71%	52	30%
**Type of hemostasis**																
Electrocautery	0	0%	0	0%	120	100%	357	99%	0	0%	0	0%	80	57%	136	67%
Ligating sutures	151	100%	218	100%	0	0%	3	1%	126	100%	251	100%	60	43%	68	33%
**Quality Score**																
Under 12	3	2%	25	12%	28	24%	145	41%	12	9%	35	14%	42	30%	2	1%
12	68	45%	85	39%	18	15%	93	25%	31	24%	112	45%	14	11%	11	5%
13	80	53%	107	49%	73	61%	120	33%	86	67%	104	41%	83	59%	191	94%
**Mean Quality Score**																
	12.50		12.29		12.18		11.56		12.54		11.56		11.84		12.24	

Table 2 also shows the score for surgical quality (13 point index) for each country and year. Quality scores in all countries were high. The mean scores across the four countries and two years ranged from 11.56 (RSA, 2012) to 12.54 (TZ, 2011). For subsequent analysis, the quality index was collapsed into three categories: under 12, 12, or 13.

The four efficiency elements and quality score were tested for their association with PPTC for each country and each year (bivariate analysis); median time for each category of the independent variable appears in Table 3. Task-shifting: TZ was the only country where both medical doctors and other clinical providers were observed to perform the operation, but doctors represented less than 10% of cases observed. No significant differences were found between provider type and PPTC. Task-sharing: PPTC was significantly lower in RSA, TZ and ZW when a secondary provider assisted in completing the operation. Number of beds: The association between number of beds and PPTC was mixed: PPTC decreased with number of beds in KE (2011) and RSA (2011, 2012), and was not related or showed no linear trend in TZ and ZW. Electrocautery: In ZW, the only country that used both types of hemostasis, using electrocautery significantly reduced PPTC. Quality score: on seven of eight data points (4 countries×2 years) there was no association between quality and PPTC; the one exception was Zimbabwe (2012), where improved quality scores were associated with increased PPTC.

**Table 3:** Median TEOT and PPTC (in minutes:seconds) for the VMMC procedure, by elements of interest and by country**.**

	Kenya				South Africa				Tanzania				Zimbabwe			
	2011		2012		2011		2012		2011		2012		2011		2012	
	TEOT	PPTC	TEOT	PPTC	TEOT	PPTC	TEOT	PPTC	TEOT	PPTC	TEOT	PPTC	TEOT	PPTC	TEOT	PPTC
**Task-shifting: cadre of primary provider**																
Physician	–	–	38∶23	12∶53	23∶03	6∶18	29∶41	6∶46	21∶38	12∶37	20∶52	11∶56	27∶20	7∶58	23∶20	6∶48
Non-Physician	22∶42	13∶34	23∶01	12∶53	30∶22	26∶01	26∶33	6∶54	24∶45	14∶06	23∶17	14∶59	–	–	–	–
Significance	τ	τ	τ	τ	τ	τ	τ	τ					τ	τ	τ	τ
**Task-sharing: who performed the suturing**																
Primary provider	22∶38	13∶35	23∶10	12∶53	19∶18	9∶10	–	–	24∶14	14∶58	23∶15	14∶59	29∶02	10∶12	20∶26	9∶23
Primary and secondary provider	35∶01	9∶11	–	–	24∶28	4∶44	29∶41	6∶46	23∶23	8∶39	22∶23	10∶51	26∶02	5∶52	24∶51	5∶41
Significance	τ	τ	τ	τ	**	**	τ	τ		**		**		**	**	**
**Number of beds**																
1	22∶45	13∶28	22∶14	12∶30	27∶47	11∶48	31∶57	12∶21	21∶32	14∶06	25∶03	15∶26	–	–	–	–
2–3	23∶28	14∶03	24∶20	14∶56	22∶24	8∶22	25∶42	6∶58	25∶54	14∶01	22∶34	13∶56	26∶31	8∶54	22∶51	6∶54
4+	17∶05	9∶10	–	–	22∶24	5∶22	30∶38	5∶51	–	–	26∶24	17∶28	27∶30	7∶33	23∶40	7∶17
Significance	**	**			**	**	**	**	**		**	**				
**Type of hemostasis**																
Electrocautery	–	–	–	–	23∶31	6∶19	29∶37	6∶43	–	–	–	–	26∶02	6∶57	23∶48	6∶02
Ligating sutures	22∶42	13∶34	23∶10	12∶53	–	–	48∶38	11∶59	24∶41	14∶06	23∶12	14∶47	27∶46	9∶10	25∶10	9∶39
Significance	τ	τ	τ	τ	τ	τ	τ	τ	τ	τ	τ	τ		**	**	**
**Quality Score**																
Under 12	24∶04	13∶40	24∶58	15∶59	26∶24	7∶25	30∶02	7∶00	26∶40	16∶37	22∶31	13∶20	25∶52	7∶33	20∶57	7∶23
12	22∶39	13∶41	22∶02	12∶12	22∶23	6∶07	29∶51	7∶19	22∶12	14∶04	23∶14	15∶19	25∶43	8∶18	21∶48	8∶00
13	22∶38	13∶28	23∶20	12∶54	22∶56	6∶14	28∶36	6∶05	23∶21	13∶08	23∶21	14∶20	29∶00	8∶36	23∶34	9∶01
Significance																
**Spearman Correlation Coefficient between Quality Score and Length of Operation**																
	−0.02	0.00	0.02	0.05	−0.09	−0.04	−0.14	−0.04	−0.11	−0.13	−0.00	0.00	0.10	0.06	0.06	0.18
Significance							**									**

* = p-value under 0.10 significance;

** = p-value under 0.05 significance; τ = cells with less than five observations were excluded from this analysis.

Bivariate analysis was also performed to test the relationship of these five explanatory variables with TEOT; see Table 3. Task-shifting: There was no relationship between task-shifting and TEOT in TZ (see caveat regarding small n in paragraph above); it was not tested elsewhere, due to insufficient cases. Task-sharing (of suturing): no clear association emerged; task-sharing was associated with increased TEOT in two cases (ZW, 2012 and RSA, 2011), and not significant in the rest. Number of beds: The association between number of beds and TEOT was mixed: TEOT decreased significantly with increased number of beds in KE (2011) and RSA (2011); TEOT increased significantly with increased number of beds in TZ (2011); and it was not related or showed no linear trend in the remaining countries. Electrocautery: In ZW, the only country that used both types of hemostasis, the use of electrocautery resulted in lower TEOT than ligaturing sutures in both years but the difference was only significant in 2012. Quality score: on seven of eight data points (4 countries×2 years) there was no association between quality and TEOT; the one exception was RSA (2012), where higher quality was associated with lower TEOT.

The results in Table 4 identify the factors significantly associated with PPTC when analyzed using multiple linear regression for each country separately. In KE there were no significant associations. The results from the other three countries showed that PPTC decreases with task-sharing in both years (except RSA 2012) and with the use of electrocautery in both years in ZW, the only country where both electrocautery and ligating sutures were used. The association between number of beds and PPTC was significant in 2012 in RSA and TZ, but the association was positive in TZ and negative in RSA. There were no significant relationships between quality of surgical technique and PPTC.

**Table 4:** Regressions by year and by country, to predict primary provider time with client (PPTC) in seconds.

	Kenya		South Africa		Tanzania		Zimbabwe	
	Coefficient	CI	Coefficient	CI	Coefficient	CI	Coefficient	CI
**Data for sites in 2011**								
**Type of hemostasis**								
Electrocautery	–	–	–	–	–	–	Ref	–
Ligating sutures	τ	–	τ	–	τ	–	**178.40** **	(92.65, 264.15)
**Task-sharing: who performed suturing**								
Primary provider	–	–	Ref	–	Ref	–	Ref	–
Primary & secondary	τ	τ	−**257.16** **	(−362.19, −152.13)	−**347.84** **	(−466.23, −227.44)	**(**−**235.53** ** **)**	(−312.67, −158.43)
**Task-shifting: cadre of primary provider**								
Physician	–	–	–	–	Ref	–	–	–
Non-Physician	τ	τ	τ	τ	0.1	(−152.21, 154.10)	–	–
**Mean number of beds**								
	−12.67	(−8.02, 32.69)	5.21	(−20.01, 30.43)	−18.37	(−97.46, 60.73)	6.22	(−10.76, 11.21)
**Mean quality score**								
	6.24	(−75.83, 88.3)	−2.42	(45.05, 40.22)	−47.7	(−107.17, 11.77)	15.15	(−6.65, 36.95)
**Data for sites in 2012**								
**Type of hemostasis**								
Electrocautery	–	–	–	–	–	–	Ref	–
Ligating sutures	τ	–	τ	–	τ	–	166.59	(100.33, 232.85)
**Task-sharing: who performed suturing**								
Primary provider	–	–	–	–	Ref	–	Ref	–
Primary & secondary	τ	–	τ	–	−**264.02** **	(−468.15, −59.89)	−**184.51** **	(−249.43, −119.6)
**Task-sharing: cadre of primary provider**								
Physician	–	–	–	–	Ref	–	–	–
Non-physician	τ	–	τ	–	−**146.00** **	(−315.18, 23.18)	τ	–
**Mean number of beds**								
	49.34	(−51.65, 150.33)	−**48.60** **	(−61.67, −35.53)	**65.42** **	(17.94, 112.90)	−16.75	(−51.33, 17.82)
**Mean quality score**								
	−39.50	(−102.6, 23.6)	4.17	(−11.35, 19.69)	−9.03	(−47.35, 29.29)	81.40	(−21.42, 183.96)

* = p<0.10;

** = p<0.05; τ = cells with less than five observations were excluded from this analysis.

The results in Table 5 identify the factors significantly associated with TEOT when analyzed using multiple linear regression for each country separately. Again, there were no significant associations in Kenya. Task-sharing: in RSA (2011) and ZW (2012), TEOT increased when secondary providers shared the task of suturing. Number of beds: in TZ (only, but both years) TEOT increased with number of beds. Electrocautery: in ZW, TEOT decreased with the use of electrocautery (on average, by 4∶42 minutes in 2011 and 2∶35 minutes in 2012). Quality score: in RSA (2012 only) quality was associated with lower TEOT.

**Table 5:** Regressions by year and by country to predict total elapsed operating time (TEOP) in seconds.

	Kenya		South Africa		Tanzania		Zimbabwe	
	Coefficient	CI	Coefficient	CI	Coefficient	CI	Coefficient	CI
**Data for sites in 2011**								
**Type of hemostasis**								
Electrocautery	–	–	–	–	–	–	Ref	–
Ligating sutures	τ	–	τ	–	τ	–	**401.90** *	(−3.24, 781.04)
**Task-sharing: who performed suturing**								
Primary provider	–	–	Ref	–	Ref	–	Ref	–
Primary & secondary	τ	–	**230.35** **	(101.02, 399.69)	−82.07	(−255.54, 91.41)	−45.43	(−407.69, 316.83)
**Task-shifting: cadre of primary provider**								
Physician	–	–	**–**	–	Ref	–	**–**	–
Non-Physician	τ	τ	τ	τ	−61.295	(−281.46, 158.87)	**–**	–
**Mean number of beds**								
	−0.09	(−82.74, 82.55)	−20.5	(−61.17, 20.15)	**119.44** *	(4.23, 231.64)	−34.95	(−87.26, 17.36)
**Mean quality score**								
	32.84	(−116.57, 182.24)	−9.136	(−77.88, 59.61)	−45.86	(−136, 44.27)	64.53	(−39.08, 168.14)
**Data for sites in 2012**								
**Type of hemostasis**								
Electrocautery	–	–	–	–	–	–	Ref	–
Ligating sutures	τ	–	τ	–	τ	–	**154.79** **	(30.85, 278.73)
**Task-sharing: who performed suturing**								
Primary provider	–	–	Ref	–	Ref	–	Ref	–
Primary & secondary	τ	–	τ	–	9.19	(−244.48, 262.87)	**339.18** **	(217.75, 460.59)
**Task-sharing: cadre of primary provider**								
Physician	–	–	–	–	Ref	–	–	–
Non-physician	τ	–	τ	–	−161.49	(−371.73, 48.75)	–	–
**Mean number of beds**								
	53.99	(−103.95, 211.93)	8.06	(−28.51, 44.62)	**81.01** **	(22.00, 140.01)	−5.65	(−70.31, 59.02)
**Mean quality score**								
	−34.44	(−113.38, 64.5)	−**60.75** **	(−103.82, 17.67)	−19.62	(−67.24, 28.01)	153.62	(−38.20, 345.44)

* = p-value under 0.10 significance;

** = p-value under 0.05 significance; τ = cells with less than five observations were excluded from this analysis.

To summarize the key results, descriptive data showed the important time savings for the primary providers (where task-shifting was not authorized) in task-sharing – having a secondary provider complete suturing – and the use of electrocautery. On 6 of 8 data points (4 countries×2 years) the median TEOT was 23–25 minutes. Multivariate results confirmed the relationship between task-sharing (having a secondary provider complete the suturing) and PPTC. Electrocautery also decreased PPTC. Factors related to TEOT varied by country and year, but there was evidence of task-sharing reducing TEOT in the two countries where task-shifting is not authorized, as well as electrocautery (in the one country where it could be tested). Quality of surgical technique was not significantly related to either PPTC or TEOT in regression analysis, except for RSA in 2012 where higher quality was associated with lower TEOT.

## Discussion

SYMMACS provides the first detailed analysis of timing of the steps in the VMMC procedure, PPTC, and TEOT, based on data from field settings from four African countries. The results demonstrated the benefits of two of the six elements of surgical efficiency: task-sharing and electrocautery. SYMMACS provided weak or inconclusive evidence on task-shifting as measured by cadre of the provider (for lack of in-country variation) and number of beds. It was not designed to test two of the six elements: surgical method and use of pre-bundled kits with disposable instruments.

The inconclusive results on task-shifting and number of beds merit additional interpretation, which further increases understanding of efficiencies in VMMC programs. The benefit of task-shifting – using well-trained clinical providers who are not doctors to complete all aspects of the procedure – relates to the greater availability and the lower cost of this cadre of provider to programs, not reduced operating time. The use of medical doctors as primary providers actually increased the median TEOT, as shown in RSA (2012) and ZW (2011), the two countries that did not authorize task-shifting to other clinical personnel. Similarly, increased number of beds *per se* does not reduce TEOT; in fact, in the case of TZ, TEOT actually increased with increased number of beds. Rather, use of multiple beds allows the primary provider – particularly important where task-shifting is not authorized – to attend to more clients in a given period of time. The time that the client spends on the operating table (TEOT) is not lower, but the time the primary provider spends with each client is reduced, increasing the overall efficiency of the program. Where space is a greater constraint then human resources, multiple beds are not likely to increase efficiency.

In the multivariate analysis, there was little evidence of a relationship between quality of surgical technique and either PPTC or TEOT (with one exception, RSA in 2012). One possible explanation relates to the clustering of results toward the high end of the quality scale: the large majority scoring 12–13 on a 13 point scale; the fact that other items on the QA assessment, reported elsewhere, were scored partially or not satisfactory adds face validity to the high scores on quality of surgical technique. The lack of association between quality and TEOT refutes the notion that reducing the time of operation in high volume setting results in decreased quality of care. Whereas SYMMACS revealed a decrease in quality of services – measured on multiple dimensions with the rapid expansion of the program (e.g., in South Africa, see Rech et al. [17] in this supplement) – this analysis demonstrated that quality did not suffer as a result of increased speed of surgical steps. On the contrary, in the only case that was significant in the multivariate analysis, higher quality was related to lower operating time (RSA in 2012), possibly as a result of experienced providers being both better and faster.

SYMMACS results showing the positive benefits of task-sharing confirm the findings of Lissouba et al. [18] for VMMC in South Africa and of Curran et al. [12] for VMMC in Kenya, which demonstrated that non-physicians could adequately perform many elements of the VMMC procedure, including suturing. The results for electrocautery are consistent with the observations of Machado et al. [19], Massarweh et al. [20], and Shen et al. [21] of its benefits in reducing operating time for the primary provider without any increase in complications in other types of surgical procedures. Others have discussed the benefit to VMMC programs of training other clinical providers (not medical doctors) as a means of lowering costs to the program while retaining quality with similar findings [22], [23]. Research on task-shifting to other clinical providers has yielded comparable results to doctors for other types of procedures: post-partum tubal ligation [24], [25], IUD insertion [26], [27], and emergency obstetric surgery [28]. The few studies that have related quality of the operation to efficiency in terms of time-savings had mixed results. Mahler et al. [29] found that it was possible to improve quality and save time for VMMC procedures through the use of multiple beds and increased provider experience, while Walker and Adam [30] reported that improving efficiency resulted in provider concerns about safety and increased likelihood of burnout in Australian hospitals.

These results should be considered with a number of limitations in mind. The number of sites visited per country varied, as a result of differences in number of functional VMMC sites per country and sampling strategy (e.g., random sample in Kenya, explained elsewhere[14]). Where possible, the same sites were visited in 2011 and 2012, resulting in some providers being included in both years; yet there was no way to identify which providers were observed twice. The non-normal distribution of the data resulted in comparison of medians rather than means, which reduced overall power. The lack of within-country variation on efficiency elements (collinearity between country and efficiency element) precluded combination of data across countries, and hence separate analyses were done for each country and by year (4countries×2years). The lack of variation within a country on efficiency elements led to a small sample size on multiple variables, precluding even within country analysis on those variables. In addition, the time variables were not normally distributed. This was addressed by using non-parametric methods in the analysis. Despite using transformation methods in the regression analysis, the times still did not have normal residual distributions. Finally, no amount of surgical efficiency can compensate for weak demand for VMMC services; operating at peak efficiency yields benefits only under high volume conditions. However, the issue of calibrating supply and demand was beyond the scope of this research.
